# Dependence on Generative Artificial Intelligence Among Medical Students and Its Association With Critical Thinking: A Cross-Sectional Study

**DOI:** 10.7759/cureus.112457

**Published:** 2026-07-11

**Authors:** Siva P M, David Sam Selva Jeyam P, Hiranmahi Gandhi K

**Affiliations:** 1 Community Medicine, Tirunelveli Medical College, Tirunelveli, IND; 2 Community Medicine, Madras Medical College, Chennai, IND

**Keywords:** artificial intelligence (ai), critical thinking, generative ai dependency, medical education, medical students

## Abstract

Introduction: Generative artificial intelligence (AI) (e.g., ChatGPT and Google Gemini) is exceptionally useful in the educational field for learning new concepts. However, besides its benefits, it also introduces the risk of pathological dependence on AI. Hence, this study aims to evaluate the prevalence of AI dependency and its association with critical thinking.

Methods: A cross-sectional study was conducted at a government medical college in Tamil Nadu. The study population comprised undergraduate medical students. Data were collected using a structured digital questionnaire that consisted of validated Generative AI Dependency and Critical Thinking Disposition Assessment Questionnaire (CTDAQ-20) scales. Statistical analysis included non-parametric group comparisons, Spearman's rank correlation, and multiple linear regression.

Results: Among the 722 participants, the prevalence of Generative AI usage was 695 (96.3%). Among users (n=695), the median AI dependence score was 2.1 (IQR: 1.6-2.7), with males having higher dependence than females (p=0.019) and final-year students reporting higher dependence than students in other years (p<0.001). The overall median critical thinking score was 71.0 (IQR: 64.0-77.0), with female students scoring higher (72 vs. 69; p<0.001), and first-year students having better critical thinking scores than students in other years (p=0.031). Overall, AI dependence had a significant inverse correlation with overall critical thinking (rho=-0.248, p<0.001), and multiple linear regression confirmed that higher AI dependence (B=-1.487, p<0.001) and increasing age (B=-0.868, p=0.008) were associated with lower critical thinking.

Conclusion: Generative AI usage is ubiquitous among medical students. However, greater dependence on AI is independently associated with diminished critical thinking, particularly among final-year and male students. Therefore, it is hypothesized that the medical curriculum should consider incorporating assessment methods that require active learning and clinical reasoning, such as objective structured clinical examinations (OSCEs) and direct observation of procedural skills (DOPS), rather than relying primarily on traditional theory-based examinations, and should promote AI literacy that supports the use of AI as an adjunct rather than a replacement for human cognition.

## Introduction

The emergence of Generative AI, including large language models such as ChatGPT, Google Gemini, and Grok AI, has revolutionized education and transformed learning practices in higher education [1-4]. Particularly in higher education and clinical medicine, these AI tools are used to learn complex concepts and retrieve information easily from vast numbers of sources [3]. Yet, the use of these AI tools acts as a double-edged sword: while helping facilitate learning and improve information literacy, it also introduces the rising phenomenon of “AI dependency.” AI dependency is defined as an excessive reliance on AI tools to perform tasks that traditionally require human cognitive effort [1].

According to the Interaction of Person-Affect-Cognition-Execution (I-PACE) model, students may use AI as a maladaptive coping mechanism to manage academic stress, maintain academic self-efficacy, and meet increasing performance expectations [1]. Excessive reliance on AI has also raised other concerns, namely "cognitive offloading," outsourcing cognitive demands to digital tools, and "cognitive inertia," a state of diminished active cognitive engagement [3].

Empirical evidence shows that students who use AI tools excessively have a significant reduction in critical thinking, the ability to objectively analyze and evaluate information to form a judgment, by sacrificing the depth of analytical inquiry for the speed of automated output [1,3]. Alarmingly, longitudinal studies in an adolescent population have shown a significant increase in AI dependency rates from 17.14% to 24.19% within six months [2].

Despite extensive literature on AI use among general university students, a critical gap remains regarding medical students, especially in India. Since the MBBS curriculum demands extensive scientific knowledge and continuous access to evidence-based data for clinical reasoning, medical students are more prone to AI dependence. This threat has raised concerns that over-reliance on these Generative AI tools may compromise diagnostic reasoning, making the quality of future patient care uncertain. Previous studies have reported problematic AI use among students and highlighted concerns about the potential adverse effects of AI dependence on critical thinking and cognitive autonomy. Although student acceptance and utilization of generative AI tools in higher education are increasing rapidly, evidence regarding their impact on medical students remains limited. Additionally, since India trains one of the largest medical student populations globally, understanding the association between AI dependence and critical thinking among them is necessary [1,3,4]. Hence, this study primarily aims to bridge this gap by assessing dependence on Generative AI among undergraduate medical students in India and its association with critical thinking ability. The secondary objectives are to estimate the prevalence and severity of Generative AI dependency, assess critical thinking skills among medical students, and identify demographic variations, thereby guiding the development of educational strategies.

## Materials and methods

Study design, setting, and duration

The study was conducted as a cross-sectional observational study at Tirunelveli Medical College, a tertiary teaching institute in Tamil Nadu, India. Data collection was carried out in April and May 2026. The study was conducted in accordance with the Strengthening the Reporting of Observational Studies in Epidemiology (STROBE) guidelines.

Study population and participant selection

The study population included first-year to final-year undergraduate medical students enrolled in the MBBS program at Tirunelveli Medical College.

Inclusion and exclusion criteria

All undergraduate medical students who provided voluntary informed consent and were willing to take part in the study were included. Students on long leave and those not willing to participate in the study were excluded.

Sample size calculation and sampling technique

The minimum required sample size was estimated using the formula (single population proportion): n=Z²pq/d². Based on the study conducted by Huang S et al., the prevalence (p) of AI dependency was taken as 24.19%, with a 95% confidence level (Z=1.96) and an absolute precision of 5% (d=0.05) [2]. The minimum required sample size was calculated as 282 participants.

However, with the objective of obtaining adequate representation from all academic years and considering the exploratory nature of the study, all eligible undergraduate MBBS students enrolled in the institution were invited to take part in the study. Participation was voluntary, and data were collected continuously throughout the study period.

**Figure 1:**
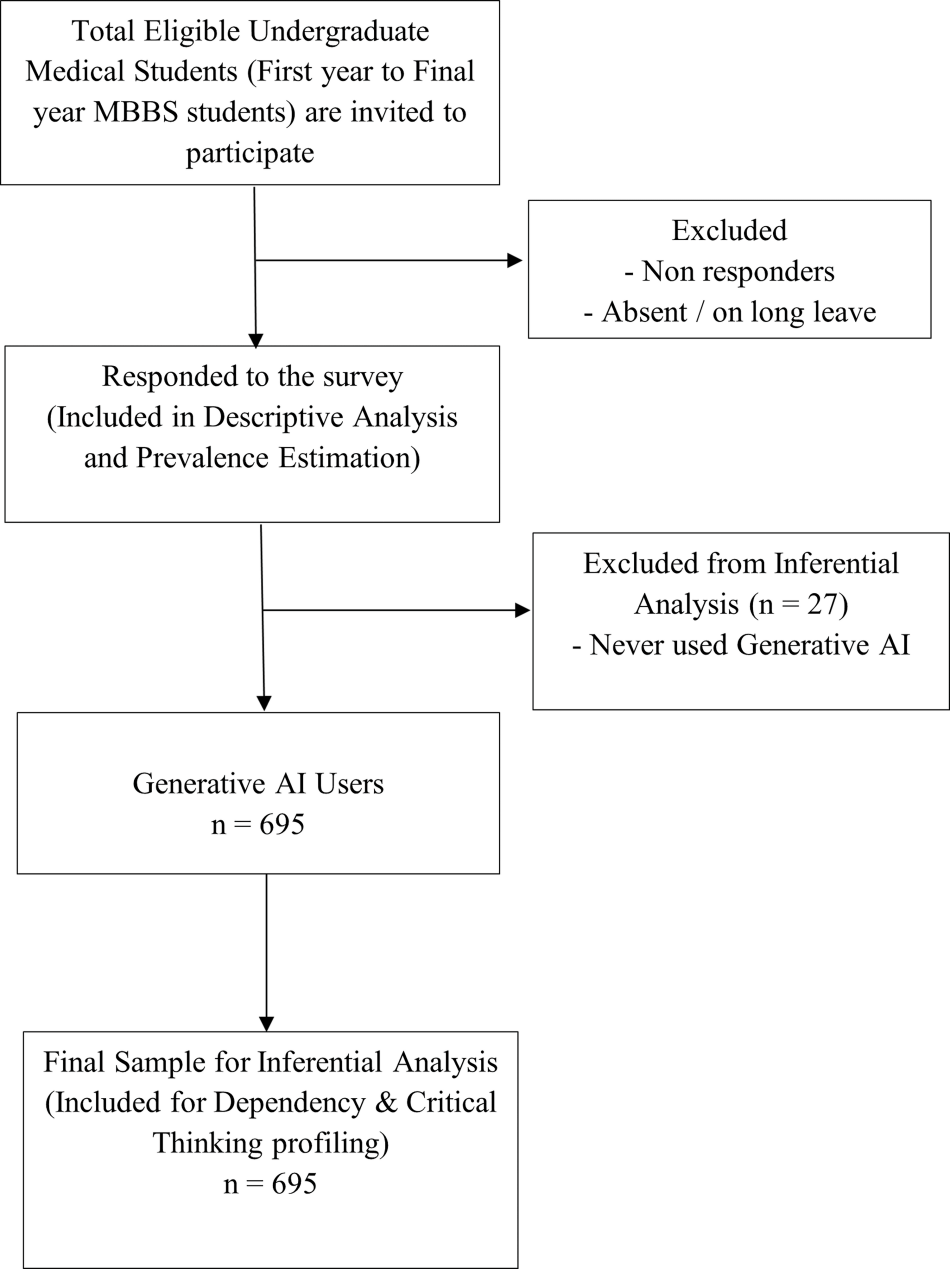
Flowchart showing the selection of study participants AI: artificial intelligence

A total of 722 out of 1000 students responded to the survey, with a response rate of 72.2%. All 722 responses were used for descriptive analysis. For inferential analysis, after excluding 27 participants who had never used generative AI tools, 695 responses were included. The final sample substantially exceeded the minimum calculated sample size, thereby improving the precision of the estimates and the statistical power for subgroup analyses (Figure 1).

Operational definitions and questionnaire used

Two validated instruments were used to structure the questionnaire used for the study, which helped evaluate the primary objective of the study.

Generative Artificial Intelligence Dependency

Generative AI dependency was assessed using the 11-item Generative AI Dependency Scale developed by Goh et al. [5]. Responses were recorded on a five-point Likert scale ranging from one (strongly disagree) to five (strongly agree). Item scores were summed and averaged according to the original scoring procedure, with higher scores indicating greater Generative AI dependency.

The Cognitive Preoccupation domain assesses the obsessive mental preoccupation with AI and its influence on daily decision-making. The Negative Consequences domain assesses functional impairments, including perceived declines in academic performance, social relationships, and mental well-being related to AI usage. The Withdrawal domain evaluates the psychological distress (e.g., anxiety and irritability) experienced when AI use is restricted [5].

The overall score is calculated by taking the mean of the scores of all three domains. A higher overall score indicates greater pathological dependency on AI tools.

Critical Thinking Disposition

The critical thinking capability was assessed using the Final Critical Thinking Disposition Assessment Questionnaire (CTDAQ-20) developed and validated by Mukhopadhyay et al., especially among Indian students, and is internally consistent (Cronbach's alpha=0.783). This is a 20-item Likert scale tool with three domains, namely: Confidence and Systematicity (7 items), Analyticity and Flexibility (9 items), and Maturity (4 items). Each item is scored on a five-point Likert scale, but items 9, 12, 14, and 15 are reverse-scored. Total scores range from 20 to 100. The higher the score, the higher the independent critical thinking capability [6].

In the current study sample, the AI Dependency Scale demonstrated excellent internal consistency (Cronbach's α=0.904; intraclass correlation coefficient (ICC)=0.904, 95% CI: 0.893-0.914) in the reliability assessment, while the Critical Thinking Scale showed good internal consistency (Cronbach's α=0.842; ICC=0.842, 95% CI: 0.824-0.858), indicating satisfactory reliability of both instruments.

The final structured questionnaire included demographic details, patterns of AI usage, and both validated scales mentioned above. It was distributed as a digital questionnaire (Google Form), and the data were collected.

The original source publications of both instruments were cited in the manuscript. Copyright of the Generative AI Dependency Scale remains with the original authors, and the instrument is published under the Creative Commons Attribution-NonCommercial-NoDerivatives 4.0 (CC BY-NC-ND 4.0) License. Permission to use the 11-item Generative AI Dependency Scale was obtained via email. Copyright of the CTDAQ-20 remains with the original authors, and the instrument is published under the Creative Commons Attribution 4.0 (CC BY 4.0) License.

Statistical analysis

Following data collection, the data were exported to the Statistical Package for the Social Sciences (SPSS) software (Version 27.0; IBM Corp., Armonk, New York) for data cleaning and further statistical analysis. Descriptive statistics were used for demographic characteristics and to estimate prevalence. Non-parametric tests were used to compare medians for the skewed data yielded from the psychometric scoring scales (AI Dependency Scale and CTDAQ-20) across demographic subgroups. The first non-parametric test used was the Mann-Whitney U test for comparing median scores of dichotomous independent groups, such as gender and residence type, residence against the AI dependency scores and critical thinking scores and their respective subdomains. The other non-parametric test used was the Kruskal-Wallis test to compare medians of independent variables with three or more categories against the scores of the psychometric scales. Another non-parametric test, post hoc pairwise comparisons with Bonferroni correction, was used to identify precisely which groups differed (in critical thinking across academic years) and to counteract the inflation of Type I errors that occurs in multiple pairwise comparisons. Effect sizes were calculated to assess the magnitude of observed differences, using effect size r (Z/√N) for Mann-Whitney U tests and epsilon-squared (ε²) for Kruskal-Wallis tests. For r, values of <0.10, 0.10-0.29, 0.30-0.49, and ≥0.50 were interpreted as negligible, small, medium, and large effects, respectively. For ε², values of <0.01, 0.01-0.08, 0.08-0.26, and >0.26 were interpreted as negligible, small, medium, and large effects, respectively.

The correlation analysis between total AI dependency and critical thinking scores was performed using Spearman's rank correlation coefficient (rho), as this test evaluates the strength and direction of monotonic relationships between the variables. This correlation was used to assess the relationship between overall AI dependency and critical thinking scores, the inter-domain relationships between the subscales of these scales, and the association between the continuous demographic variable (age) and critical thinking scores. Beyond bivariate associations, a multiple linear regression model was used to examine the relationship between a single continuous dependent variable (critical thinking score) while controlling for multiple independent variables (AI dependency score, age, gender, year of study, and residence). Throughout the analysis, the precision of inferential analysis was quantified using a 95% confidence interval (CI), particularly for Spearman's rank correlation, as the coefficients were calculated using Fisher's transformation.

## Results

A total of 722 participants were included in the descriptive data analysis. The study population ranged in age from 17 to 25 years, with a mean age of 20.31±1.46 years. The sample had a female predominance of 407 (56.4%). The distribution across all academic years was relatively even, with the highest proportion in the first year, 215 (29.8%), and the lowest in the final year, 132 (18.3%). Most participants were from urban areas, 443 (61.4%), and most were hostellers, 589 (81.6%) (Table 1).

**Table 1:** Sample characteristics, demographics (n=722), and prevalence of generative AI use ^#^Prevalence of Generative AI usage in the current sample population. IQR: interquartile range; SD: standard deviation; AI: artificial intelligence

Characteristic	n/mean	% or SD
Age (years)		
Mean±SD	20.31	1.46
Range	17-25	
Gender		
Male	315	43.6
Female	407	56.4
Year of study		
First year	215	29.8
Second year	169	23.4
Third year	206	28.5
Final year	132	18.3
Accommodation		
Day scholar	133	18.4
Hosteller	589	81.6
Residence		
Urban	443	61.4
Rural	279	38.6
Generative AI usage		
Yes	695	96.3^#^
No	27	3.7
AI usage duration (months)		
Mean±SD	15.41	10.64
Median (IQR)	12.0	(6.0-24.0)

The prevalence of Generative AI use among the study population was exceptionally high, with 695 participants (96.3%), with a median duration of AI use of 12 months (IQR: 6 to 24 months). Regarding the patterns of AI usage, over half of the participants, 378 (54.4%), used generative AI daily, while 179 (25.8%) used it more than two to three times a week. The primary purpose reported by most participants, 674 (97.0%), was using Generative AI for academic purposes, specifically for concept clarification, 580 (86.1%), writing support, 375 (55.6%), and achieving a deep understanding of subjects, 365 (54.2%) (Table 1).

Generative artificial intelligence dependence

The overall median AI dependence score was 2.1 (IQR: 1.6-2.7) among Generative AI users. Domain-specific analysis showed that the Negative Consequences domain had the highest median score of 2.5 (IQR: 1.8-3.0), followed by the Cognitive Preoccupation domain with a median score of 2.0 (IQR: 1.3-3.0) and the Withdrawal domain with the lowest median score of 1.5 (IQR: 1.0-2.3).

Significant demographic variations were observed in Generative AI dependency scores. Compared with females, male students had higher AI dependency (p=0.019), particularly in the Cognitive Preoccupation (p=0.016) and Withdrawal (p<0.001) domains. However, the observed gender differences were of negligible to small magnitude (effect size r=0.089-0.149). AI dependence also varied significantly across academic years (p<0.001), although the effect size was small (ε²=0.024). Post hoc pairwise comparisons using Bonferroni correction showed that final-year students had significantly higher dependence than first-year students (adjusted p=0.001). Cognitive Preoccupation and Withdrawal also differed significantly across academic years, with small effect sizes (ε²=0.038 and 0.015, respectively), whereas Negative Consequences did not differ significantly (p=0.124, ε²=0.004) (Table 2).

**Table 2:** Association of socio-demographic profile with overall and domain-specific generative AI dependence scores (N=695) ^*^Mann-Whitney U test, ^†^Kruskal-Wallis test. IQR: interquartile range; Cog. Preocc.: Cognitive Preoccupation; Neg. Cons.: Negative Consequences; r:effect size for Mann-Whitney U test, ε^2^: effect size for Kruskal-Wallis test; AI: artificial intelligence

Variable	Total dependence (median, IQR)	Test value, effect size, p-value	Cog. Preocc. (median, IQR)	Test value, p-value	Neg. Cons. (median, IQR)	Test value, p-value	Withdrawal (median, IQR)	Test value, p-value
Male	2.2 (1.7-2.8)	U=53406.5^*^, r=0.089, P=0.019	2.3 (1.7-3.0)	U=53313.5^*^, r=0.091, P=0.016	2.5 (2.0-3.0)	U=61033.0^*^, r=0.021, P=0.584	2.0 (1.0-2.8)	U=49635.5^*^, r=0.149, P<0.001
Female	2.0 (1.5-2.6)		2.0 (1.3-2.7)		2.5 (1.8-3.3)		1.3 (1.0-2.0)	
First year	1.9 (1.4-2.5)	H=19.541^†^, ε^2^=0.024, P<0.001	1.7 (1.3-2.3)	H=28.753^†^, ε^2^=0.038, P<0.001	2.5 (1.8-3.0)	H=5.766^†^, ε^2^=0.004, P=0.124	1.3 (1.0-2.0)	H=13.257^†^, ε^2^=0.015, P=0.004
Second year	2.1 (1.6-2.9)		2.0 (1.3-3.0)		2.5 (2.0-3.3)		1.8 (1.0-2.5)	
Third year	2.2 (1.7-2.8)		2.2 (1.7-3.0)		2.5 (2.0-3.3)		1.8 (1.0-2.5)	
Final year	2.3 (1.7-2.9)		2.3 (1.7-3.0)		2.8 (2.0-3.0)		2.0 (1.0-2.8)	
Urban	2.1 (1.6-2.7)	U=55676.5^*^, ε^2^=0.014, P=0.708	2.0 (1.3-3.0)	U=53548.5^*^, ε^2^=0.046, P=0.225	2.5 (1.8-3.3)	U=55722.5^*^, ε^2^=0.014, P=0.720	1.5 (1.0-2.5)	U=58473.5^*^, ε^2^=0.028, P=0.459
Rural	2.1 (1.6-2.6)		2.0 (1.3-3.0)		2.5 (2.0-3.0)		1.8 (1.0-2.3)	

Critical thinking

The overall median critical thinking score of the participants was 71.0 (IQR: 64.0-77.0). Among the domains, the Analyticity and Flexibility domain yielded the highest median score of 33.0 (IQR: 29.0-36.0), and the Maturity domain yielded the lowest median score of 12.0 (IQR: 11.0-14.0).

Comparison across demographic characteristics showed that female students had significantly higher overall critical thinking scores than male students (72 (IQR: 67-77) vs. 69 (IQR: 61-76), p<0.001), although the magnitude of this difference was small (r=0.165). Similar small effect sizes were observed across all critical thinking domains (r=0.119-0.159). Overall critical thinking scores also differed significantly across academic years (p=0.031), with first-year students having the highest median score of 72 (IQR: 67-78) and final-year students having the lowest median score of 69 (IQR: 60-78). However, this difference was associated with a negligible effect size (ε²=0.008). Likewise, Maturity demonstrated a statistically significant but small effect across academic years (ε²=0.010), whereas conversance and systematicity and Analyticity and Flexibility showed no significant differences (p=0.377 and 0.056, respectively). No significant differences in critical thinking scores or domain scores were observed according to residence, with all effect sizes being negligible (r=0.002-0.037) (Table 3).

**Table 3:** Association of sociodemographic characteristics with overall and domain-specific critical thinking scores among generative AI users (N=695) ^†^Kruskal-Wallis test, ^*^Mann-Whitney U test. r: effects size for Mann-Whitney U test; ε²: effects size for Kruskal-Wallis test; AI: artificial intelligence; IQR: interquartile range

Variable	Critical thinking score, median (IQR)	Test value, p-value	Conversance and systematicity, median (IQR)	Test value, p-value	Analyticity and Flexibility, median (IQR)	Test value, p-value	Maturity, median (IQR)	Test value, p-value
Gender		U=71041^*^, r=0.165, P<0.001		U=70186.0^*^, r=0.153, P<0.001		U=67834.5^*^, r=0.119, P=0.002		U=70455.0^*^, r=0.159, P<0.001
Male	69 (61-76)		25 (21-27)		32.5 (28-36)		12 (11-13)	
Female	72 (67-77)		26 (24-28)		34 (30-37)		13 (12-14)	
Year of study								
First year	72 (67-78)	H=8.846^†^, ε²=0.008, P=0.031	26 (23-28)	H=3.098^†^, ε²=0.000, P=0.377	34 (30-37)	H=7.575^†^, ε²=0.007, P=0.056	13 (12-14)	H=9.931^†^, ε²=0.010, P=0.019
Second year	70 (65-77)		25 (23-27)		33 (29-36)		12 (11-14)	
Third year	71 (63-76)		25 (22-27.5)		33 (29-36)		12 (11-14)	
Final year	69 (60-78)		25 (21-28)		33 (27-36)		12 (11-13)	
Residence								
Urban	71 (64-77)	U=55156.5^*^, r=0.022, P=0.563	25 (22-28)	U=54814.0^*^, r=0.027, P=0.475	33 (29-36)	U=56502.5^*^, r=0.002, P=0.958	12 (11-14)	U=54158.5^*^, r=0.037, P=0.327
Rural	71 (65-76)		25 (22-27)		34 (29-36)		12 (11-14)	

Correlation of age and artificial intelligence dependency with critical thinking and its domains

Age showed a weak negative correlation with overall critical thinking scores (ρ=-0.150, p<0.001). The AI dependency score was inversely correlated with overall critical thinking (ρ=-0.248, p<0.001) and all critical thinking domains, with the strongest association seen in Maturity (ρ=-0.277, p<0.001). Cognitive Preoccupation was negatively associated with Confidence and Systematicity (ρ=-0.136), Analyticity and Flexibility (ρ=-0.145), and Maturity (ρ=-0.257) (all p<0.001). Similar negative associations were seen for Negative Consequences and Withdrawal. Among the domains of the AI Dependency Scale, Withdrawal showed the strongest correlations with Analyticity and Flexibility (ρ=-0.224) and Maturity (ρ=-0.262), suggesting that greater dependence-related symptoms were associated with poorer critical thinking performance (Table 4).

**Table 4:** Correlation of age and generative AI dependency with overall and domain-specific critical thinking scores among generative AI users (N=695) ρ: Spearman's correlation coefficient; AI: artificial intelligence

Correlation analysis	Critical Thinking Scale			
	Critical thinking score	Conversance and systematicity	Analyticity and Flexibility	Maturity
Age	ρ=-0.150, p<0.001	ρ=-0.118, p=0.002	ρ=-0.143, p<0.001	ρ=-0.080, p=0.036
Generative AI Dependence scale				
Overall, AI dependence score	ρ=-0.248, p<0.001	ρ=-0.185, p<0.001	ρ=-0.198, p<0.001	ρ=-0.277, p<0.001
Cognitive Preoccupation	ρ=-0.194, p<0.001	ρ=-0.136, p<0.001	ρ=-0.145, p<0.001	ρ=-0.257, p<0.001
Negative Consequences	ρ=-0.157, p<0.001	ρ=-0.123, p=0.001	ρ=-0.118, p=0.002	ρ=-0.200, p<0.001
Withdrawal	ρ=-0.266, p<0.001	ρ=-0.194, p<0.001	ρ=-0.224, p<0.001	ρ=-0.262, p<0.001

Multiple linear regression analysis predicting critical thinking score

The regression model was statistically significant (F=9.845, p<0.001) and explained 6.7% of the variance in critical thinking scores (adjusted R²=0.060). Higher AI dependency scores independently predicted lower critical thinking scores (B=-1.487, β=-0.128, p=0.001). Increasing age was also associated with lower critical thinking scores (B=-0.868, β=-0.138, p=0.008). Female gender independently predicted higher critical thinking scores (B=2.794, β=0.150, p<0.001). Year of study and residence were not significant predictors after adjustment (Table 5).

**Table 5:** Multiple linear regression analysis predicting critical thinking score among medical students (N=695) B: unstandardized regression coefficient; SE: standard error

Predictor	B	SE	Standardized β	95% CI for B	P-value
Constant	86.806	6.102	-	74.826 to 98.785	<0.001
AI Dependence Score	-1.487	0.434	-0.128	-2.340 to -0.635	0.001
Age	-0.868	0.325	-0.138	-1.507 to -0.230	0.008
Gender	2.794	0.698	0.150	1.423 to 4.164	<0.001
Year of study	0.217	0.439	0.025	-0.645 to 1.080	0.621
Residence	-0.165	0.718	-0.009	-1.575 to 1.245	0.818

## Discussion

The primary aim of the study was to evaluate the epidemiological distribution of Generative AI dependence and its effect on critical thinking capability among undergraduate medical students. The study findings revealed near-universal adoption of Generative AI tools (prevalence: 96.3%, n=695) by students, particularly for academic purposes such as concept clarification and writing support. Notably, this study revealed that higher AI dependency is associated with lower critical thinking ability, as confirmed by multivariate regression analysis, posing a potential threat to the cognitive autonomy of medical students needed in the current evidence-based medical field and to the diagnostic reliability of future doctors. Although the inverse correlation between AI dependence and critical thinking was statistically significant, the effect size was modest (ρ=-0.248), suggesting that critical thinking may also be affected by other factors alongside AI dependence, which warrants further study.

Previous studies have reported that AI dependency is associated with psychological distress and academic performance among university students, and the high prevalence of AI usage observed in our study is consistent with findings from recent multicenter studies among medical students [7-9]. Our finding that AI dependence is negatively correlated with all domains of critical thinking, especially the Maturity and Analyticity and Flexibility domains, supports concerns regarding the impact of Generative AI on cognitive autonomy and the phenomenon of "cognitive offloading" [3,10,11]. However, these correlations were uniformly weak in magnitude, suggesting that although the relationship is consistent across cognitive domains, the decline in critical thinking cannot be attributed solely to AI dependence. This may lead students to sacrifice the depth of their autonomous, independent analytical thinking in favor of the speed of automated outputs, resulting in "cognitive inertia" [1,3].

The I-PACE model provides a plausible explanation for this behavior, suggesting that students may rely on AI tools as a coping mechanism to manage academic stress and maintain expectations for academic performance. Similar findings have been reported among university students, where higher AI dependency was associated with psychological distress and academic performance-related factors [1,7].

Subgroup analysis of demographic variations revealed significant findings. Male students showed significantly higher AI dependency, especially in the Cognitive Preoccupation and Withdrawal domains, and lower overall critical thinking scores than their female counterparts. Nevertheless, the corresponding effect sizes for gender differences were negligible to small (r=0.089-0.165), indicating that while these differences were statistically significant, their practical magnitude was limited. Additionally, AI dependency increased across academic years, peaking in the final year, which also had the lowest critical thinking scores. The differences across academic years were associated with small effect sizes (ε²=0.015-0.038 for AI dependency domains and ε²=0.008 for overall critical thinking), suggesting only modest variation attributable to academic progression. This trend may be driven by the academic demand for more evidence-based data and information literacy across a spectrum of medical subfields for final examinations, compelling students to rely heavily on AI and rapid information-retrieval tools. However, their corresponding lower critical thinking scores raise concerns that they may be at risk of reduced diagnostic reasoning and decision-making ability. Although the observed difference in AI dependence remained significant after post hoc analysis, year of study was not an independent predictor of critical thinking in the multivariable regression model, suggesting that the lower critical thinking scores among final-year students may be influenced by factors beyond academic progression alone. Nevertheless, because of the cross-sectional design, the temporal relationship between AI dependence and critical thinking cannot be established. There is also the possibility that students with lower baseline critical thinking skills are more likely to depend on Generative AI, or that sustained reliance on AI may gradually reduce opportunities to practice independent reasoning. Longitudinal studies are therefore required to determine the direction and causality of this relationship.

Addressing this challenge requires more structured interventions like objective structured clinical examinations (OSCEs) and direct observation of procedural skills (DOPS), which are more patient-based and require more active learning and reasoning than theoretical examinations. Prohibiting Generative AI in medical schools is neither feasible nor possible, as these tools have proven utility in improving information retrieval, learning efficiency, and conceptual understanding when used appropriately [10,12,13]. Therefore, assessment methodologies and curricula should evolve to emphasize clinical reasoning and independent judgment rather than focusing solely on final grades and scores in university examinations. Recent evidence from a systematic review further suggests that AI-generated educational content may influence the development of critical thinking skills among medical students, highlighting the need for responsible integration of AI into medical education [14]. Given the modest effect sizes observed in this study, educational strategies should focus on promoting balanced and reflective AI use rather than restricting AI altogether.

This study has certain limitations. The cross-sectional design precludes establishing causality between Generative AI dependence and critical thinking, and the single-center setting may limit the generalizability of the findings. Additionally, the use of self-reported questionnaires may be subject to recall and response biases. Although the observed associations were statistically significant, their consistently weak-to-small effect sizes indicate that the practical impact of AI dependence on critical thinking is modest and that critical thinking is influenced by multiple interacting educational, personal, and environmental factors. Factors such as the institutional learning environment, teaching methodology, digital literacy, and students' internet usage patterns were not evaluated and may have acted as residual confounders. Therefore, multicenter longitudinal studies are warranted to further validate these findings.

## Conclusions

Use of Generative AI was nearly universal among undergraduate medical students, with varying levels of dependence observed. Higher AI dependence was independently associated with lower critical thinking scores after adjustment for demographic characteristics, while female students exhibited comparatively stronger critical thinking dispositions. The observed associations were modest, and the regression model accounted for only a small proportion of the variability in critical thinking, indicating that multiple educational and learner-related factors likely contribute. The cross-sectional design limits causal inference. These findings indicate that AI dependence is one of several factors associated with critical thinking, rather than its primary determinant. Our findings suggest that educational approaches emphasizing clinical reasoning and independent judgment merit further investigation as potential strategies to support critical thinking in the era of Generative AI. Longitudinal multicenter studies incorporating additional educational, behavioral, and institutional factors are required to clarify the long-term relationship between AI dependence and critical thinking during medical training.
